# Apps Seeking Theories: Results of a Study on the Use of Health Behavior Change Theories in Cancer Survivorship Mobile Apps

**DOI:** 10.2196/mhealth.3861

**Published:** 2015-03-27

**Authors:** Deborah Vollmer Dahlke, Kayla Fair, Y Alicia Hong, Christopher E Beaudoin, Jairus Pulczinski, Marcia G Ory

**Affiliations:** ^1^Texas A&M School of Public HealthHealth Promotion and Community Health SciencesTexas A&M UniversityCollege Station, TX, TXUnited States; ^2^Texas A&M UniversityDepartment of CommunicationsCollege Station, TX, TXUnited States

**Keywords:** mobile apps, health behavior, survivorship, health promotion, eHealth, mobile health

## Abstract

**Background:**

Thousands of mobile health apps are now available for use on mobile phones for a variety of uses and conditions, including cancer survivorship. Many of these apps appear to deliver health behavior interventions but may fail to consider design considerations based in human computer interface and health behavior change theories.

**Objective:**

This study is designed to assess the presence of and manner in which health behavior change and health communication theories are applied in mobile phone cancer survivorship apps.

**Methods:**

The research team selected a set of criteria-based health apps for mobile phones and assessed each app using qualitative coding methods to assess the application of health behavior change and communication theories. Each app was assessed using a coding derived from the taxonomy of 26 health behavior change techniques by Abraham and Michie with a few important changes based on the characteristics of mHealth apps that are specific to information processing and human computer interaction such as control theory and feedback systems.

**Results:**

A total of 68 mobile phone apps and games built on the iOS and Android platforms were coded, with 65 being unique. Using a Cohen’s kappa analysis statistic, the inter-rater reliability for the iOS apps was 86.1 (*P*<.001) and for the Android apps, 77.4 (*P*<.001). For the most part, the scores for inclusion of theory-based health behavior change characteristics in the iOS platform cancer survivorship apps were consistently higher than those of the Android platform apps. For personalization and tailoring, 67% of the iOS apps (24/36) had these elements as compared to 38% of the Android apps (12/32). In the area of prompting for intention formation, 67% of the iOS apps (34/36) indicated these elements as compared to 16% (5/32) of the Android apps.

**Conclusions:**

Mobile apps are rapidly emerging as a way to deliver health behavior change interventions that can be tailored or personalized for individuals. As these apps and games continue to evolve and include interactive and adaptive sensors and other forms of dynamic feedback, their content and interventional elements need to be grounded in human computer interface design and health behavior and communication theory and practice.

## Introduction

We tend to overestimate the effect of a technology in the short run and underestimate the effect in the long run. 

Roy Amara, leader at the Institute for the Future

### Background

The use of mobile phones has shifted from voice and text only to the Internet accessibility of smartphones. In this shift, a large market of mobile software apps has emerged. As of May 2014, the United States had 345.2 million mobile subscribers [1]. This is more than one mobile subscription per person, based on US population estimates of 313.9 million [2]. According to the Pew Internet and American Life Surveys, 91% of US adults own a cell phone and 60% use their phone to access the Internet [3].

Mobile technology, smartphones, and tablets provide anytime anywhere access to health information, health promotion, and behavioral interventions. Use of mobile technology for health-seeking information is high, with 31% of smartphone owners using the device to search for health information [3]. Personal mobile apps are a critical component of mHealth, providing educational resources, decision-making tools, psychosocial communication, and social support.

For the growing population of cancer survivors, who experience differing needs in terms of medical care, psychosocial support, and practical needs of daily living, mHealth apps have the potential to provide access to information and health behavior interventions that are low cost, easy to access, and personalized to their specific needs. Increasingly, socially disadvantaged populations including racial/ethnic minorities, those with lower incomes, and elderly persons use mobile phones as their primary or only connection to the Internet [4,5]. While sparse, studies such as those by Bender et al are beginning to explore the efficacy and potential of mobile app interventions [5]. With relation to disease-specific apps, previous reviews have coded cancer apps to examine which apps were scientifically/clinically based or evidence-based or on the basis of the app’s purpose and content such as awareness, cancer treatment information, fundraising, or early detection [6-8]. To date, none of the research on mHealth cancer apps has systematically assessed the extent to which cancer survivorship apps, as health behavioral interventions, are theory-based.

### Study Aims

A sophisticated taxonomy of health behavior theories and behavior change frameworks developed by Abraham and Michie was later refined as a system to code Internet interventions associated with health behavior by Webb et al [9-11]. Our review further adapts that taxonomy to mHealth interventions for cancer survivorship and investigates if and how behavior change theories, some of which form the groundwork for human computer interactions and cognitive psychology, are being used. By doing so, we begin to answer two important theoretical and applied questions: “To what extent are apps for health promotion and disease prevention based on health behavior and communication theories and frameworks?” and “How can mHealth cancer survivorship apps be designed differently to be more effective health behavior change interventions?”

As a secondary aim, we considered the comparison of the degree to which the health behavior and communication theories appear to be taken into consideration based on the type of mobile platform (ie, iOS compared to Android) the apps used.

## Methods

### Overview

In preparation for this research and a number of other health communication and mobile app projects, we worked closely with over 30 cancer survivors from various racial, ethnic, age, and sociodemographic groups. We considered a cancer survivor to be any person who has been diagnosed with cancer from the time of diagnosis through the balance of life.

Prior to and during this research, we consistently received feedback on their informational needs and preferences in the use of mobile apps. In this study, we used such qualitative input in the formative stage. In November 2013, we conducted a computerized search for mHealth cancer survivorship apps on the Apple App Store for iPhone and iPad apps and on Google Play for Android apps. We explored other mobile app markets including those for Nokia, Microsoft, and Blackberry smartphones but found no cancer apps that met our criteria of being more than badges or skins. As a result of this preliminary analysis, we limited our search to native apps—software apps that must be installed on a device such as a smartphone, iPad, or table—available either for the iOS or Android platform or both. The apps could have elements or portions linked to websites or cloud-based servers, including assessments, videos, PDFs, or other linked materials, but the user interface must be initiated on the smartphone or tablet.

### Inclusion and Exclusion Criteria

Mobile app searches were conducted on Google Play and the Apple App Store using the search terms cancer + survivor, cancer + survivorship, cancer + care, cancer + treatment, and cancer + management.

Web-based searches for mobile apps were also made on Google, Bing, and Yahoo, as these are among the top search engines used in English. Search terms included cancer + mobile web, cancer survivorship + mobile web, and cancer survivorship app.

**Inclusion criteria for survivorship apps**

Inclusion criteria

- Includes specific mention of cancer care, treatment, survivorship, or cancer survivors
  Mention can be made in the description found on the app store, in the website listing, or in the table of contents of the app or its navigation terms/icons
- Applicable to one or more cancer types or cancer care
  Includes functionality and services for all cancers, information for one or more types of cancers, or information specifically addressing the late effects of cancer survivorship as a condition
- Designed for patients and survivors
  Can also include information for caregivers and providers
- Free and available for public download and use
  Input from cancer survivors suggested a reluctance to pay for apps based on the availability of free educational and instructional resources
- Offers some level of interaction for health behavior change

**Exclusion criteria for survivorship apps.**

Exclusion criteria

- Designed for research studies only
- Relevant only to patients and survivors of a specific institution or cancer center (branding of a cancer center in an app was considered allowable)
- Designed solely for use by providers
- Paid apps
- Mobile phone screen skins or badges
- Fundraising only apps
- Glossaries and mobile versions of periodicals and websites

### Study Design

The taxonomy for behavior change techniques used in this research study was derived from a taxonomy of 26 health behavior change techniques (HBCTs) by Abraham and Michie with a few important changes based on the characteristics of mHealth apps [9-11]. The mHealth app taxonomy was limited to 15 HBCTs with each described by one or more health behavior or communication theories. An additional area of HBCTs included in the mHealth taxonomy but not found in the taxonomy of Abraham and Michie and the works by Webb et al is tailored health communications (THC) [9-11]. As indicated by Rimmer and Kreuter, THCs are important elements of health communication and persuasion and may promote action through increased relevance and motivation to process information actively [12].

The theoretical models and frameworks initially used by Abraham and Michie and Michie et al [9,11] and Webb et al [10] that were included in the mHealth survivorship app taxonomy are as follows: elaboration likelihood model (ELM) (Petty and Cacioppo), social cognitive theory (SCogT) (Bandura), information-motivation-behavioral skills model (IMB) (Fisher and Fisher), control theory (CT) (Carver and Scheier), and operant conditioning (OC) (Skinner). Also used were theories related to the impact of social support on health behaviors (SS) (Cohen) and social comparison (SC) (Festinger) and theory of planned behavior (Ajzen) [12-19].

### Statistical Analysis

A coding manual was developed specifically for use in coding the mHealth apps for cancer survivorship (Multimedia Appendix 1) based on the work of Michie et al, *A Taxonomy of Behavior Change Techniques Used in Interventions* [9]. A coding guide (Table 1) drawn from the mHealth cancer survivorship taxonomy of HBCTs and theories was developed, analyzed, and tested by the coders DVD, KF, JP. The team tested and trained with the coding guide using three apps that existed both on iOS and Android mobile platforms that were not specifically related to cancer. These apps were not included in the research assessment.

Master lists of identified iOS and Android apps were developed collectively by the coders for use in downloading the apps to their smartphones and tablets. Two coders were assigned to each type of app platform, and each coder independently loaded the apps that met the eligibility criteria onto one or more mobile devices. The coding rubric used a score of 1 to indicate that the HBCT was present and 0 to indicate that the HBCT was absent. Based on two raters for each app, total possible scores for the platforms are 72 for iOS apps (36 apps times 2 raters) and 64 (32 apps times 2 raters) for the Android apps. Several apps were not working and could not be loaded and a few crashed consistently, thus preventing coding. The only major difference in coding approach was related to coding of games, and that issue was easily resolved by consensus.

**Table 1:** mHealth cancer survivorship taxonomy for coding.

Behavior Change Techniques	Theory Basis	Definition
Personalized	THC^a^, SCogT^b^, ELM^c^	Rimer and Kreuter define personalization and tailoring as a process for creating individualized communications by gathering and assessing personal data, (ie, logging in with personal information)
Tailoring (macro/meso/micro)	THC, ELM	Macro occurs at the group level; meso is determined by individual needs of user but is not highly specific; micro is very specific to the user
Health behavior linkage	IMB^d^	General information about linkage of individual behavior and health (ie, benefits of good nutrition and physical activity)
Action/behavior consequences	TRA^e^, TPB^f^, SCogT,IMB	Information about potential benefits and costs of action or inaction in relation to health and well-being (ie, stop smoking)
Intention formation	TRA, TPB, SCogT, IMB	Encourage the person to take an action or decide on a goal to improve treatment response or survivorship
Provide instruction	SCogT	Show or tell the user how to perform a behavior (ie, asking your doctor questions)
Provide materials for education	SCogT	Provide information or educational materials about cancer care and survivorship
Goal setting	CT^g^	Prompt specific goal setting (ie, walk 5 miles daily)
Self-efficacy	SCogT	Aid user in recognizing skills or education developed
Feedback on performance	CT	Scores, tests, game results
Persuasion (general/targeted)	OC^h^	Messages to strengthen self-efficacy/control beliefs
Social influence: information on peer behavior (passive)	SCogT	Facilitate user access to information on how others have changed behavior or addressed challenges (nonexpert)
Opportunity for social comparison (active)	SS^i^/SC^j^	Facilitate active user engagement in social media for sharing and comparison
Mobilize social norms (exposure to important others)	SS/SC	Provide user exposure to expert opinions and information

^a^THC, tailored health communication model

^b^SCogT, social cognitive theory

^c^ELM, elaboration likelihood model

^d^IMB, information-motivation-behavioral skills model

^e^TRA, theory of reasoned action

^f^TBP, theory of planned behavior

^g^CT, control theory

^h^OC, operant conditioning

^i^SS, social support

^j^SC, social comparison

## Results

### Ratings for Health Behavior Techniques

**Figure 1:**
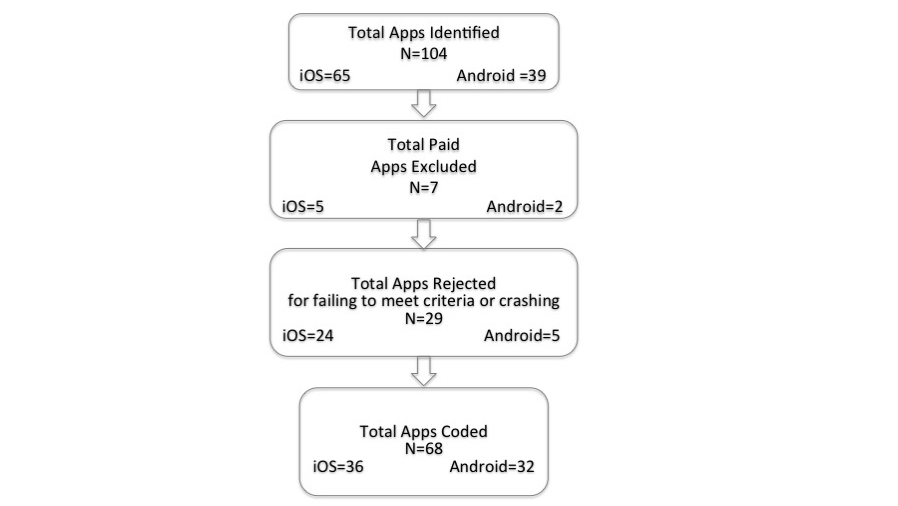
Android and iOS app selection flow chart.

A search of the mHealth cancer survivorship apps yielded a total of 104 potentially relevant apps that appeared to meet the selection criteria. After removing the paid apps (N=7) and those that crashed or would not open (N=29), there were 68 apps that were coded. A flow diagram showing the numbers, source, and refinement of the apps identified for coding is shown in Figure 1.

There were three apps that were available on both the Apple App Store and Google Play, and both teams coded these apps. Seven of the Android cancer apps were configured as games, as were four iOS games. A total of 68 unpaid apps were coded and 65 of these were unique. Using a Cohen’s kappa analysis statistic, the inter-rater reliability for the iOS apps was .86 (*P*<.001) and for the Android apps it was .77 (*P*<.001). Table 2 shows the results of the teams’ scorings of the HBCT for both Android and iOS platforms.

In considering our secondary aim of comparing platforms, we found that the iOS cancer survivorship apps received higher scores across nearly all of the HBCT areas. Additionally, the iOS apps appeared to have greater functionality and appeared to include more of the HBCTs overall, per app, than the Android apps.

**Table 2:** Rating totals for health behavior change techniques. Each item was scored as 1=present or 0=not present. Total possible scores for each platform are 72 (2×36) for iOS apps and 64 (2×32) for Android apps based on the use of two raters for each type of app.

Techniques/Characteristics	iOS HBCT Scores (N=36)	Android HBCT Scores (N=32)
Personalization	48	24
Tailoring, macro	45	15
Tailoring, meso	8	6
Tailoring, micro	11	4
Health behavior linkage	32	32
Action/behavior consequences	21	2
Prompt for intention formation	48	10
Provide instruction	54	10
Provide materials for education	28	12
Prompt for specific goals	10	14
Review of goal activity	2	2
Self-monitoring of goals	24	13
Feedback/evaluation of goals	18	16
General persuasion	25	2
Tailored persuasion	10	0
Social influence (passive)	17	0
Social influence (active)	18	8
Social norms—opportunity for comparison to important others	8	1

### Percentages of Health Behavior Change by Category

The percentage of HBCTs for each category of both the iOS and the Android platforms is shown in Table 3. A discussion of the percentages and interpretation of the results is found in the section following Table 3.

**Table 3:** Category and platform percentages for health behavior characteristics. Each item was scored as 1=present or 0=not present.

Technique/Characteristic	iOS Platform, % (N=36)	Android Platform, % (N=32)	Total for Both Platforms, %
Personalization	67	38	53
Tailoring, macro	63	23	44
Tailoring, meso	11	9	10
Tailoring, micro	15	6	11
Health behavior linkage	44	50	47
Action/behavior consequences	29	3	11
Prompt for intention formation	67	16	25
Provide instruction	75	16	32
Provide materials for education	39	19	30
Prompt for specific goals	14	22	18
Review of goal activity	3	3	3
Self-monitoring of goals	33	20	27
Feedback/evaluation of goals	25	25	25
General persuasion	35	3	20
Tailored persuasion	14	0	7
Social influence (passive)	24	0	13
Social influence (active)	25	0	19
Social norms—opportunity for comparison to important others	11	2	6

**Figure 2:**
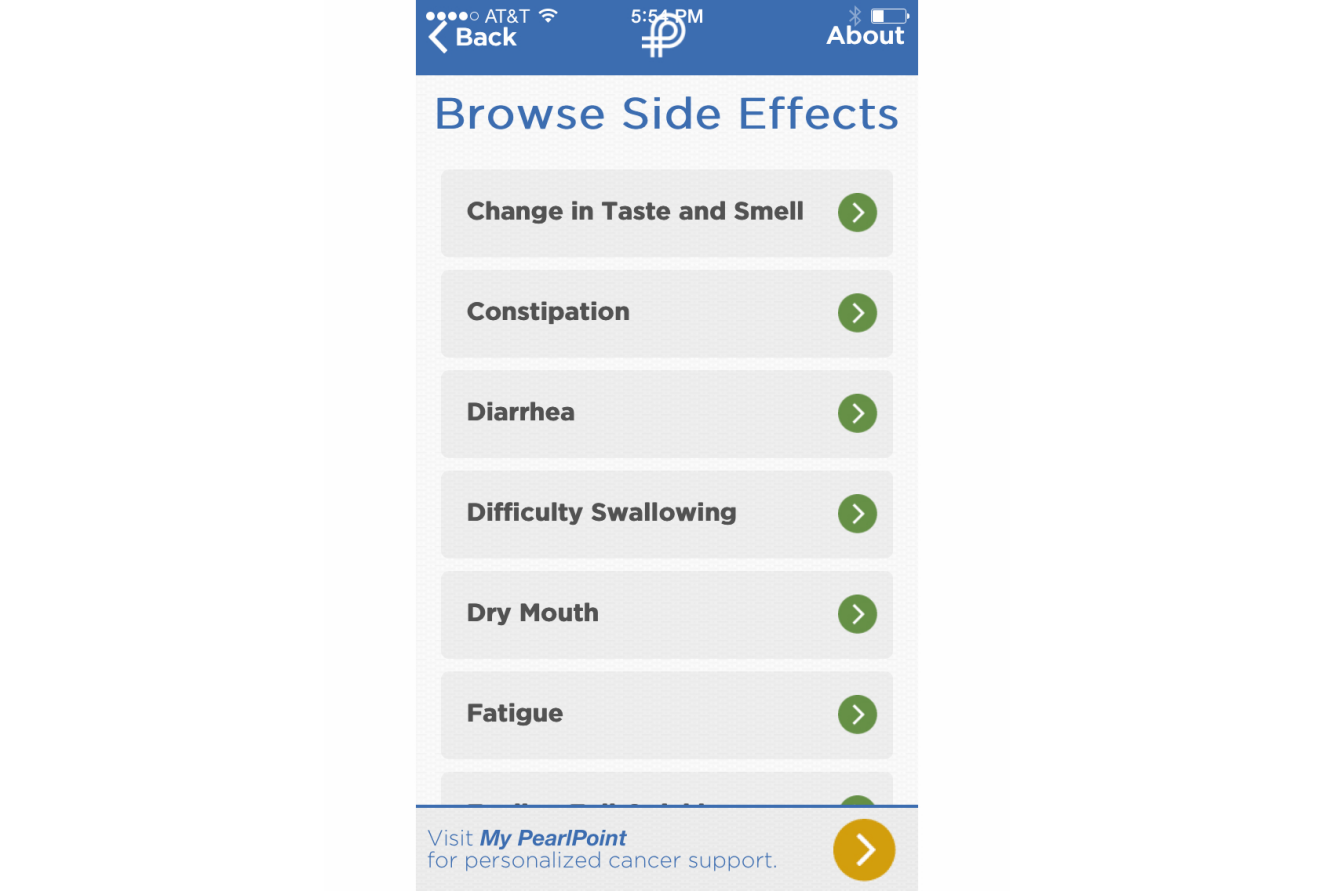
My PearlPoint app.

Personalization in the apps includes requiring that the user log in with a username and password and was present in 67% of the iOS apps (48/72) and 38% (24/64) of the Android apps. For most of the apps, personalization enabled access to selected parts of the app and also allowed data to be entered and maintained on the app’s server rather than being stored on the phone, thus providing adequate security for sensitive health information. Several apps requested specific information about the user’s type of cancer and then provided meso- or micro-level tailoring regarding concerns such as types of treatment and late effects. Macro-tailoring was the most commonly found technique with 63% (45/72) for iOS and 23% (15/64) for Android. An example of personalization (Figure 2), with both macro- and meso-level tailoring, is found in the *Cancer Side Effects Helper* app developed by PearlPoint Cancer Support. The app allows users to identify the side effects they may be experiencing (eg, fatigue, dry mouth, nausea). Once a user selects a side effect, the app provides education and health behavior linkages and may also suggest specific goals or actions to reduce the identified side effect.

Scoring on health behavior linkages was indicative of the app providing basic information about cancer care and survivorship, including diagnosis, treatment, and/or availability of resources for clinical or non-clinical purposes. Based on the high scores for this HBCT on both iOS and Android apps, it appears that most of the apps, 94% (64/68), provide a basic level of health behavior information.

iOS apps had much higher scores for action/behavior consequences at 29% (21/72) as compared to the Android platform apps at 3% (19/64). Scoring for this category indicates that the app provides information or feedback on health behavior changes suggested or stimulated by the app.

**Figure 3:**
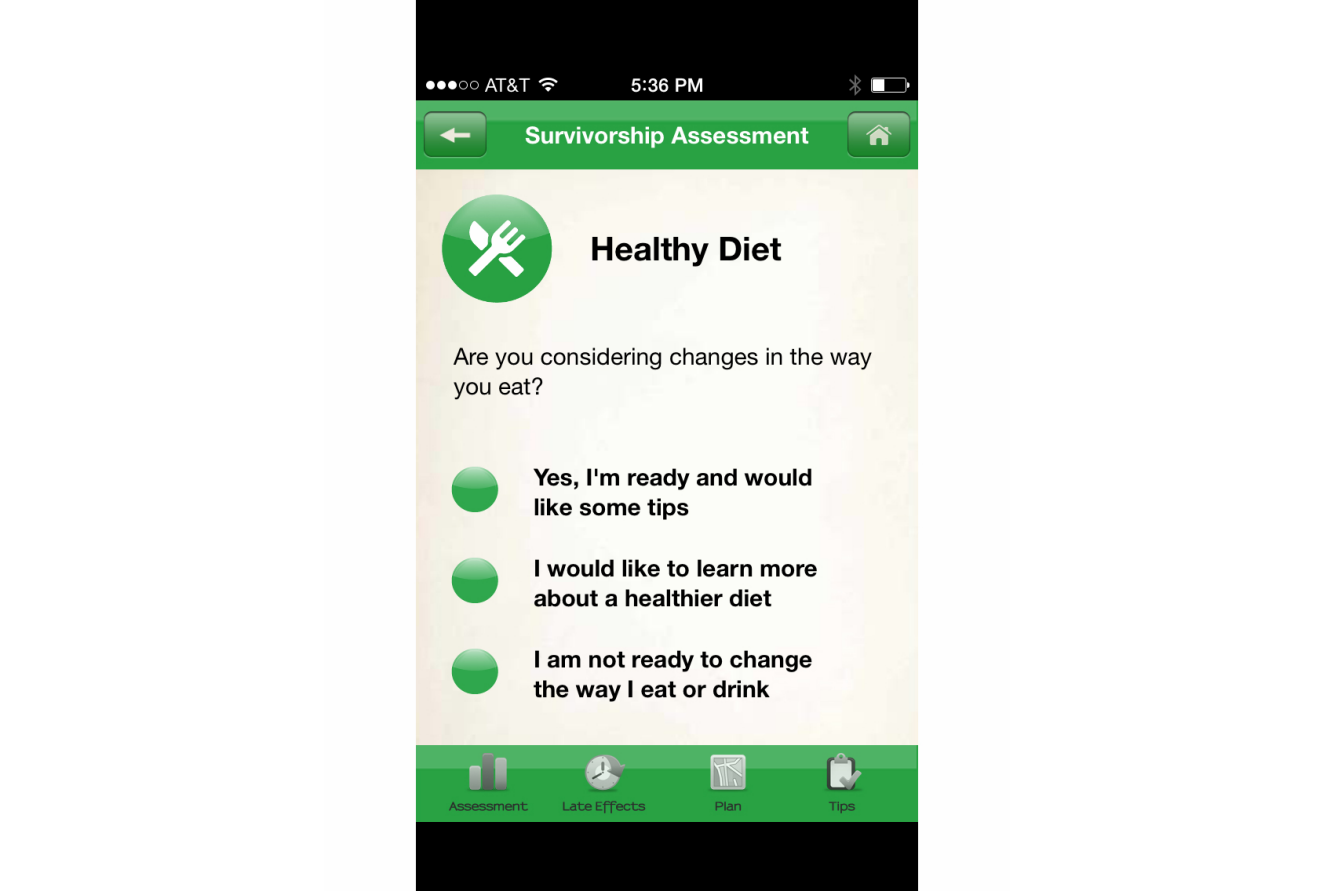
AYA Healthy Survivorship app: intent formation.

The prompt for intention formation HBCT was coded as positive if the app included suggestions for general behavior or for formulating desired outcomes of a behavior for healthy survivorship (eg, maintain a healthy weight, exercise daily, stop smoking, and consider medication). The scoring of the apps indicated that iOS apps at 67% (48/72) included this technique more frequently than Android apps at 16% (10/64). This HBCT concerns the user’s intent to do something and is different from taking the step to set a goal or initiate an action. Such behavioral intention is a critical motivational factor in determining whether a person actually adopts a behavior, as discussed by Ajzen [17]. An example of prompting for intent formation can be found in the *AYA Healthy Survivorship* app, an iOS app, shown in Figure 3.

**Figure 4:**
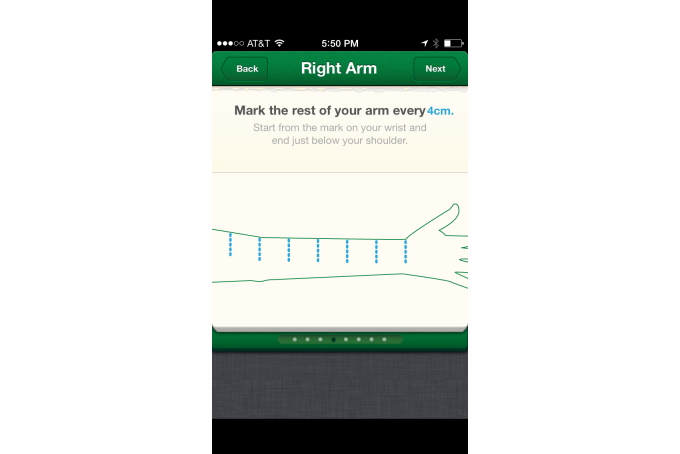
Lymphedema Tracker app.

The iOS app *Lymphedema Tracker,* shown in Figure 4, provides instruction on how to measure a survivor’s arm to set a baseline and do ongoing measurements to track lymphedema. Following the trend, a greater percentage of the iOS apps (54/72, 75%) provided instruction on this HBCT as compared to the Android apps (10/64, 16%).

**Figure 5:**
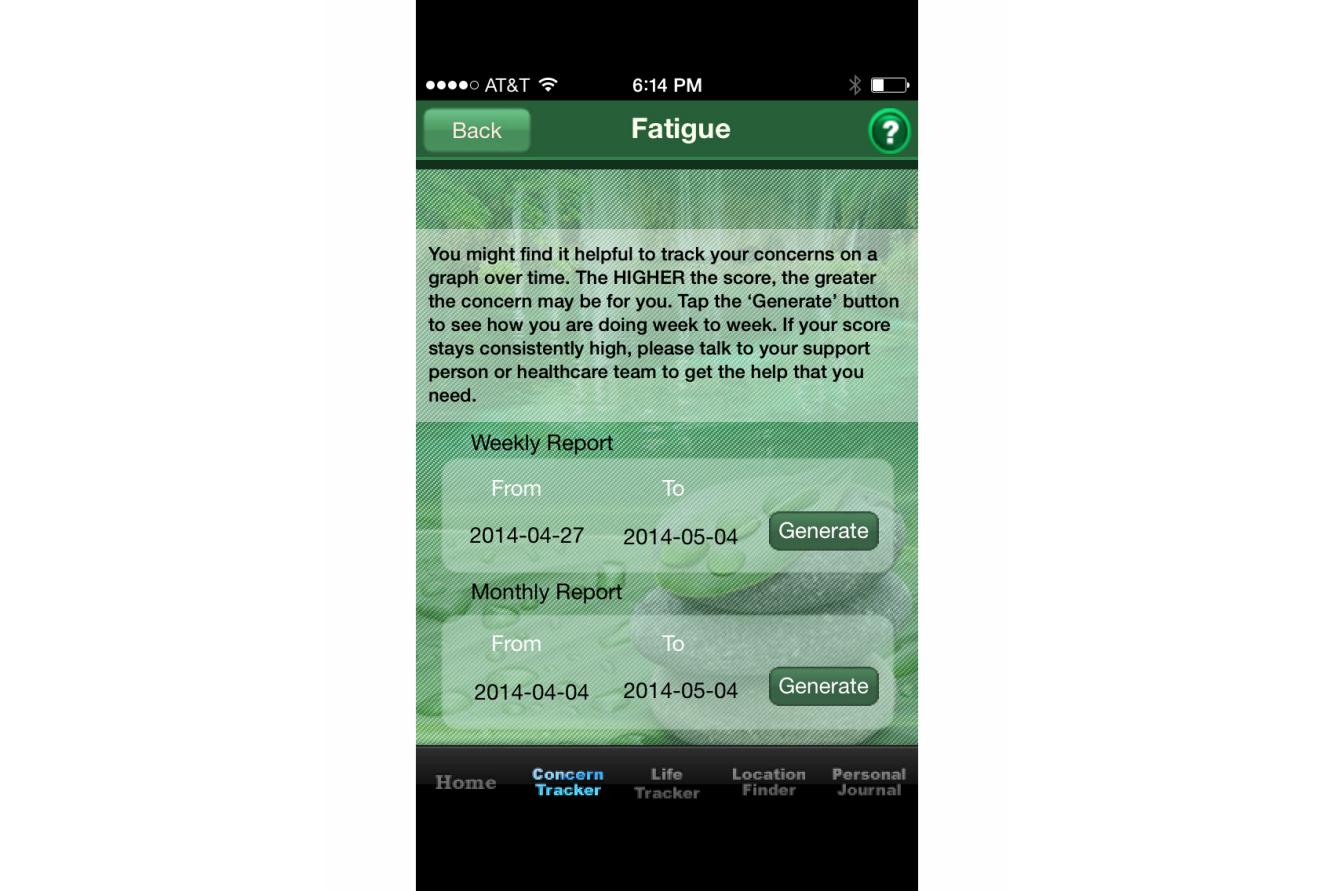
My Cancer Manager app.

A significantly greater percentage of the iOS apps (28/72, 39%) demonstrated the provide materials for education HBCT when compared to the Android platform apps (12/64, 19%). To be coded as positive, the material on the app had to be directly related to showing or telling the user ways to facilitate a specific health behavior change. An example of education specific to managing fatigue, a common concern for survivors and in post-treatment, is found in the iOS app *My Cancer Manager* from the Cancer Support Community (Figure 5). The app educates patients on the activity of tracking their fatigue and also instructs them to consider sharing information with a provider if the score stays persistently high. Overall the app scores for this HBCT were low at 39% (28/72) for the iOS apps and 19% (12/64) for the Android apps.

The presence of activities or information across both iOS and Android platform apps for goal-setting activities (eg, prompts for specific goals, review of goals, self-monitoring of goals, and feedback or evaluation of goals) was low overall. Examples in the area of self-monitoring of goals were suggestions found in several apps for the survivor to record brief notes or keep a diary or journal to record behaviors and actions related to health behaviors. Examples found among the apps included journals or tracking tools for pain and distress monitoring, as well as suggestions for practicing meditation. Among the iOS apps, these categories ranged from a low of 3% (2/72) for reviews of goal activity to a high of 33% (24/72) for self-monitoring of goals. Similarly, but lower still, the Android apps ranged from a low of 3% (2/64) to a high of 25% (16/64). These low scores included activities in the mHealth cancer survivor game apps for engaging in a first-person shooter cancer-destroying activity with goals for hitting targets. The user generally received feedback on scores for numbers of strikes or targets acquired. Other apps prompted goal setting via use of guided imagery suggesting that the user focus energy and concentration on specific body parts or processes affected by cancer.

**Figure 6:**
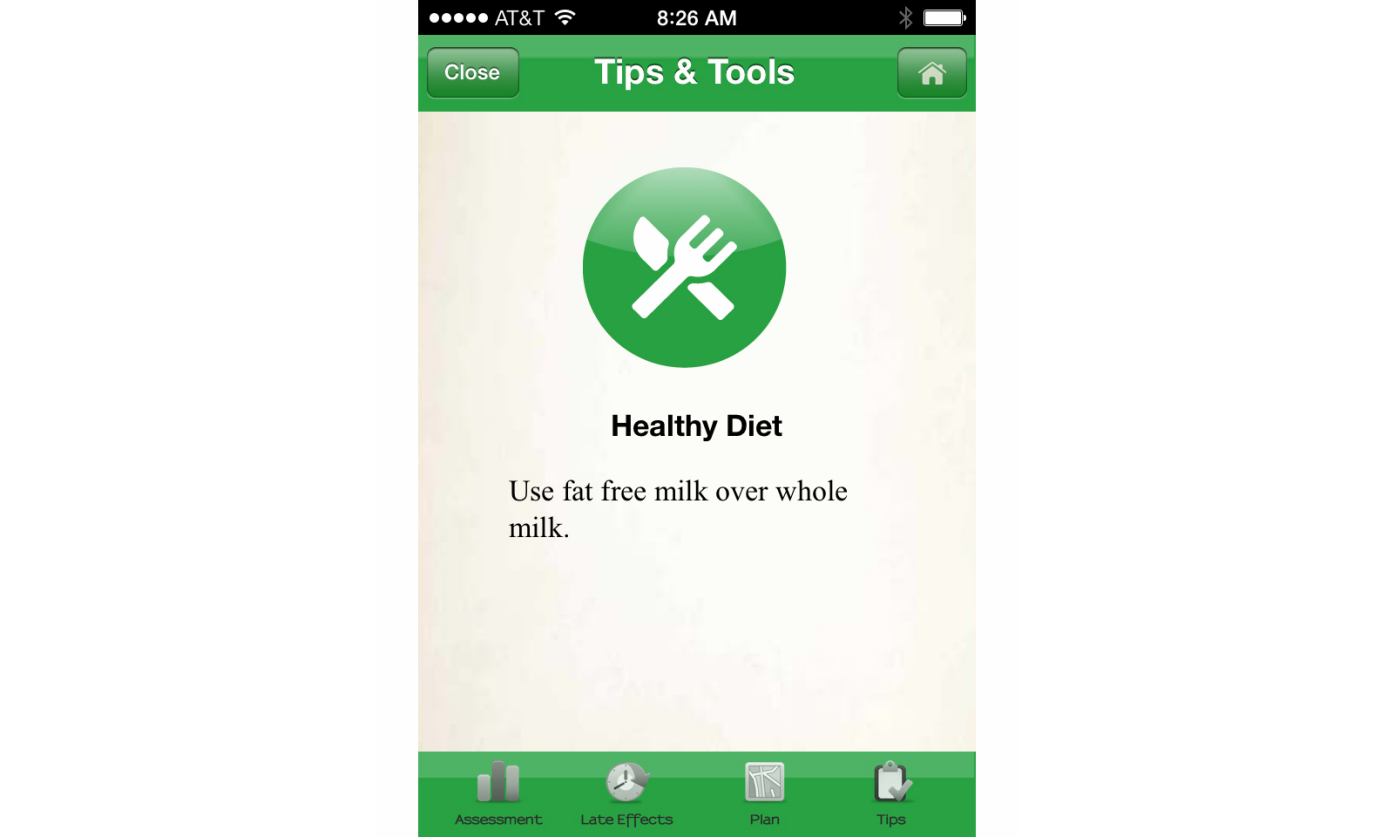
AYA Healthy Survivorship app: daily tip.

The delivery of personalized or tailored messages designed to strengthen efficacy/control beliefs related to the initiation or execution of health behavior change has been heralded as an area of promise for mHealth apps. The use of mHealth persuasion in cancer survivorship apps includes activities or signaling for new beliefs and or new information. Scores in this area were low for both general and targeted persuasion. As an example, Figure 6 shows use of general persuasion that can be found in the *AYA Healthy Survivorship* iOS app that allows the user to elect to receive a “health tip of the day.” The highest scores were found among the iOS apps, with a low for targeted persuasion of 14% (10/72) and a high of 35% (25/72) for general persuasion. Persuasion barely registered as an HBCT area on the Android apps with a low of zero for tailored persuasion and 3% (2/64) for general persuasion.

Social influence is an area of HBCT techniques that would appear to be a strong opportunity area for mHealth apps in cancer survivorship, given the easy access to mobile communities including Twitter, Facebook, YouTube, and numerous other cancer-related blog and social networking sites. The presence of passive social influence, where the app provides stories, anecdotes, interviews, or case histories about what other cancer survivors have done or experienced was, again, unexpectedly low. For the iOS apps, only 24% (17/72) offered access to such stories and Android apps had no scores for this HBCT. Active social influence, wherein a survivor might be invited to participate in a group or peer discussion and relay activities about their own, also was relatively low with a score of 25% (18/72) for iOS and 0 for the Android apps.

**Figure 8:**
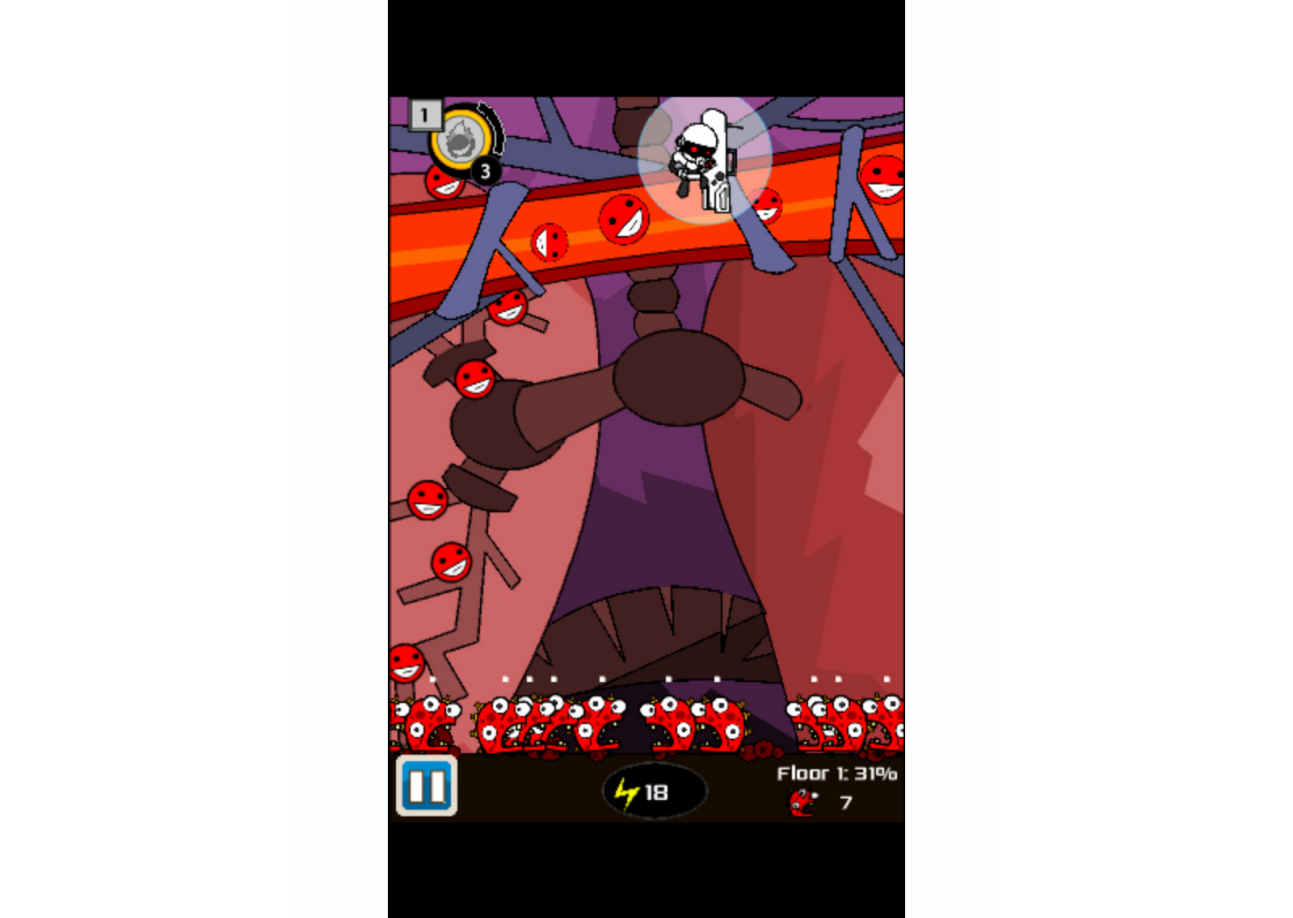
Re-Mission 2: Nanobot's Revenge app.

The apps were examined for examples of mobilization of social norms in which the user would be exposed to the social norms of important others in relation to a healthy survivorship activity or health behavior change. Important others could include a valued and trusted expert such as a health care professional or a celebrity cancer survivor advocate. One of the apps that uses this HBCT most effectively is the *Cancer.net* app (Figure 8), which supports RSS feeds on the app linking users to physicians and important role models. Cancer.Net was developed by the American Society of Clinical Oncology and is offered on both the iOS and Android platforms.

*Cancer.net* also provides the user with links for brief videos of well-respected cancer researchers and clinicians on a range of topics, including HBCTs. While this is an area of HBCT that offers easy access on mHealth apps, few of the apps reviewed in the study incorporated this potentially important element. Scores for these apps were 11% (8/72) for iOS apps and 2% (1/64) for Android apps. The evidence base for considering highly interactive mobile games and interactive game-like elements for guided imagery in apps is very limited. The potential applications of health behavior change theories in the design of game and game-like interventions are significant, ranging from elements of personalization and tailoring for scoring to goal setting, tracking and feedback, and potentially powerful elements of social interaction among game participants.

### Games for Cancer Survivorship

**Figure 7:**
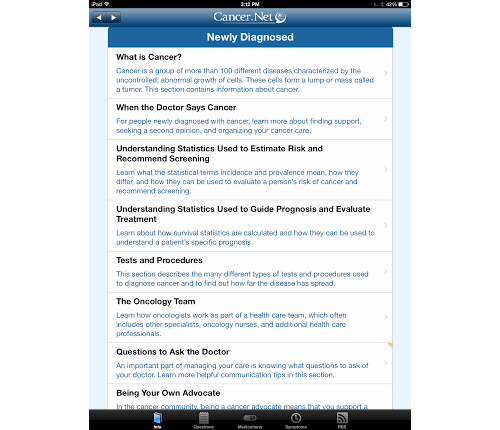
ASCO's Cancer.Net app.

Our team was initially optimistic about the inclusion and rating of the four iOS and Android mHealth games and interactive apps for cancer survivorship. App names such as *Cancer Fighter, Whip Cancer,* and *Play to Cure* promised much but delivered little as HBCT interventions. Few of the interactive or game apps provided even basic education or information for HBCTs. Rather, the user was launched into series of images and audio effects with opportunities to score by shooting down images on the mobile screen but offered little or no explanation about what might be of benefit to the cancer survivor. An exception was found in *Re-Mission2: Nanobot’s Revenge* (Figure 7), a project of HopeLab that initiates the shooter game by explaining that the user is the Nanobot and the goal is to “fire targeted treatment at growing cancer and prevent it from escaping into the blood stream.”

## Discussion

### Principal Findings

A primary aim of this study was to analyze the linkage of HBCT interventions in cancer survivorship mHealth apps to theories and models that are used to predict health behavior change and communications, including those that are specific to information processing and human computer interaction such as control theory and feedback systems. The mHealth apps in this study varied greatly in how they ranked in the use of theoretical elements of health behavior change. This study’s findings are consistent with prior research that asserts that mHealth interventions could benefit from increased use of behavior and communications theories in their design [10-16,20,21]. In reviewing the HBCT scores for the apps, three theories/models appeared to be most influential: SCogT, THC, and CT. However, with no explicit discussion regarding the design or development of the apps reviewed in this study, it is not clear if these theories were intentionally applied or that the design deliberately reflected a theoretical approach. Moreover, the HBCT elements were just barely present in the game apps, which made up 16% (11/68) of all coded apps.

**Figure 9:**
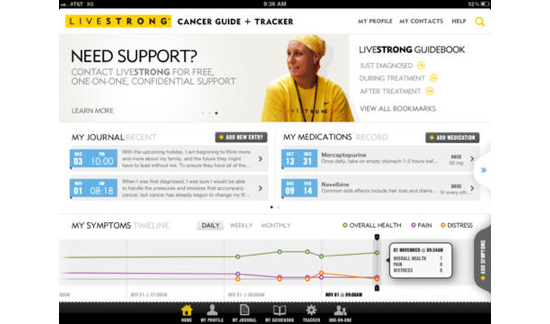
Livestrong Cancer Guide and Tracker app.

The mHealth cancer survivorship apps that appeared to be firmly based in HBCT theory were similar in that they offered multiple types of HBCTs, required personalization and some degree of tailoring, were highly interactive, included some type of questions or assessments, suggested goals and actions, and provided social engagement and the mobilization of social norms. Most of these examples were either developed by cancer advocacy groups, clinical associations, or academic researchers, which suggests that the information provided was more likely to be based in evidence and clinical research and health behavior theory. Examples include (1) Livestrong *Cancer Guide and Tracker* app, available only for iPad (see Figure 9); (2) *Cancer.net*, developed by the American Society of Clinical Oncology, available on iOS and Android platforms (see Figure 8) and also offers a Web-based version and one that is translated into Spanish; (3) *AYA Healthy Survivorship*, an iOS app, developed for Adolescent and Young Adult (AYA) survivors by Texas A&M School of Public Health with late effects guidelines provided by the Children’s Oncology Group (see Figures 4 and 6); and (4) *My Cancer Manager* developed by the Cancer Support Community and available only as an iOS app (see Figure 5).

An area of HBCT that demonstrated weak results in the coding of the mHealth apps considered in this study but one that bears additional research consideration is the theoretical realm of social influence and social media. Cancer survivors are strongly influenced by their social ties and connections with others, and social networks can have important effects on survivor health and wellbeing [4,8,22].

### Strengths and Limitations of This Study

The strength of this study is based in its reliance on the prior work of Abraham, Webb and Michie in defining the taxonomy and coding of behavior change techniques used in interventions and their basis in theory [9-11]. This research on mHealth cancer survivorship apps had certain limitations. The initial search and selection of cancer survivorship apps was restricted to the commercial descriptions of apps available for Microsoft, Nokia, Blackberry, Apple, and Android phones. Based on that review and the application of our criteria, we narrowed our search to the Apple App Store and Google Play. Based on input from a variety of cancer survivors, we included only unpaid apps, although we did examine the paid apps to see if they appeared to include greater HBCT in their design. They did not. The search results for this study were dependent on the terms included in the search strategy and the functionality of the search engines used. We attempted to overcome the search limitation by choosing common terms and combinations of terms, including those we found in literature reviews of cancer care and cancer survivorship. We considered only apps that were in English. Moreover, we only considered apps that were focused on cancer and included the word cancer in the title or the description. It is possible that we missed apps that include cancer care and survivorship in addition to other chronic diseases.

### Future Directions in App Development

Clearly, the taxonomy provided in this research for mHealth cancer apps is not exhaustive, and additional theories and models across different behavioral change techniques should be defined. As the more highly rated apps in our study offered multiple HBCT techniques, it may be beneficial to design a study that takes into account the interaction across multiple HBCT aspects. It may also be helpful to explore the differences in use, HBCT efficacy, and persistence on the device for single purpose apps for specific survivorship concerns in comparison to apps that offer multiple types of HBCT elements. Research and exploration into the theories and models relevant to interactive apps, mobile games, and the use of sensors in mHealth is timely and needed.

An article by Tomlinson et al further articulates concerns about both the lack of evidence and theory in mHealth and how theory, when referenced, is actually applied. Tomlinson’s article addresses use of mHealth primarily in lesser-developed and under- resourced countries but raises concerns about level of evidence and generalizability of mHealth apps [23]. A World Health Organization report by Kay and associates that tracked over 500 mHealth pilot studies reported that very little is known about likely uptake, best strategies for engagement, efficacy, or effectiveness of these initiatives [24]. Kay and colleagues’ review of mHealth interventions suggests that the apps they reviewed lacked both theoretical foundations and evidence sufficient to support an evidence-based scale-up. The most recent systematic review on mHealth for health behavior change by Free et al was not able to identify the theoretical basis for the research studies reviewed [22]. Both reviews confirm that there is mixed evidence for the effectiveness of health intervention delivery to health care consumers using mobile technologies. Moreover, both reviews’ conclusions highlighted the need for additional high-quality controlled trials of mHealth apps. These findings regarding the potential for use of theory support a call for mHealth intervention designers to reflect more deeply and extensively on the application of theoretical models and frameworks in the design and development of mobile HBCT apps.

### Conclusions

The study provides a framework for future research and contributes to the emerging science of mHealth interventions for behavior change. The findings suggest a strong rationale for investing the time and diligence into more rigorous theory-based mHealth interventions that may incorporate, as did the apps reviewed, multiple levels and types of health behavior change strategies and techniques. Similarly, our results reinforce the need for carefully constructed studies to measure the effect and impact of mHealth interventions.

Our findings contribute to behavioral health literature and health policy initiatives by demonstrating that mHealth intervention design needs stronger theoretical and evidence-based underpinning. The field of research on mHealth interventions for behavior change is rapidly shifting with new technologies and systems for sensors, big data analytics, and opportunities for more patient-centric health care. The integration of apps with mobile hardware, including sensors, and electronic medical records is rapidly emerging as evidenced by the ongoing announcements of such integrated solutions. While the promise of interoperability of apps, sensors, and clinical data will soon be a reality, what is missing is the understanding of how this will translate into benefits to users with chronic medical conditions, such as cancer survivors. What is also missing is how, where, and when clinicians will access and use this data to educate, inform, and offer improved opportunities for health and wellness to their patients.

## Multimedia Appendix 1

Survivorship App Coding Manual.

## Abbreviations

CT: control theory
ELM: elaboration likelihood model
HBCT: health behavior change techniques
IMB: information-motivation-behavioral skills model
OC: operant conditioning
SC: social comparison
SS: social support
SCogT: social cognitive theory
THC: tailored health communications
