# Supplementary material for: Apps Seeking Theories: Results of a Study on the Use of Health Behavior Change Theories in Cancer Survivorship Mobile Apps
Source: JMIR Mhealth Uhealth. 2015 Mar 27;3(1):e31. doi: 10.2196/mhealth.3861 (PMC4393507; doi:10.2196/mhealth.3861)
Supplement: Supplementary file 1 [file mhealth_v3i1e31_app1.pdf]

Coding Manual for mHealth Behavioral Change Techniques in Cancer Survivorship Applications

2014

(An adapted version of the coding manual from Abraham C and Michie S. A Taxonomy of Behavior Change Techniques use in Interventions: The Coding Manual. (2007)

General Instructions for Coding Mobile Health Applications

Please review the mHealth theory and behavior change taxonomy before coding the mobile health applications (mHealth apps). Discuss the techniques with co-coders to ensure that all coders interpret these materials, definitions, and techniques similarly. Conduct at least one practice coding session jointly with practice materials that are comparable (but different from) the final study materials and discuss these results with the team before beginning coding the study applications. The following suggestions assume that the selection of the mHealth apps has already been made and that there is agreement among the team that all of the selected apps meet the stated criteria for inclusion in the study.

Suggestions for optimal coding of health behavior mhealth apps:

- Read all selection and coding material before coding.
- Scan the different health behavior techniques presented in the coding table or Coding Scoring Sheet as these may differ by different types of apps.
- Print out the Behavior Change Theory Definition Matrix and one Coding Scoring Sheet for each mHealth application. Put the name and source of the mHealth Application on the Coding Scoring Sheet
- Start coding using the Coding Scoring Sheet. In case of any doubts between the techniques, please re-read the descriptions in the Theory Analysis and Definition Matrix

After you have finished coding a mHealth application, please review the completed coding scoring sheet to make sure that you have scored the correct techniques and that you have crossed out any techniques that are not use in the mHealth app under consideration.

Request: If you have suggestions for improvements or extensions of this coding manual please make them prior to beginning to code. The coding manual is a work in progress, but for consistency in the study all coders need to begin and end with the same set of instructions.

General Techniques in mHealth Applications

Each scoring sheet has specific areas to score or annotate the presence of personalization, tailoring and participation.

1.Personalization: This is the provision of opportunities in the mHealth application to make elements of the application personal by the selection of tools or elements that are specific to the individual using the application. An example would be the ability to select a disease type from among several available in the application and then to follow a specific path or set of tools or systems. For example, being able to select “breast cancer” and then being provided sets of information specific to that type of cancer. Another example would be the ability to select to receive emails or texts of a specific nature. The choice of “yes” or “no” to a specific capability of the application would be considered personalization.

2. Tailoring: Coders are asked to annotate the score sheet for each mHealth application to indicate the app’s capability to include an intervention element or component that is specific to the characteristics of the person using the app. Coders will be asked to score tailoring at three different levels in the initial assessment of the mHealth application (see Coding Scoring Sheet):
1) Macro-tailoring at the group level. In this instance the mHealth application can be adapted to adjust the intervention materials (including information) that the participant receives based on pre-tested characteristics. For example an app may ask the user if he/she wishes to receive texts and/or assessments on diet, on exercise or smoking cessation.
2) Meso-tailoring at the individual level. The amount or type of intervention depends on the individual needs of the participant. For example, the participant could select between texts delivered once a day versus once a week.
3) Micro-tailoring at the individual level. Specific techniques in the mHealth application are tailored to the unique individual. For example personalized goal setting and reporting tailored to the individual’s own needs and desires

for physical activity. Or GPS tracking and reporting of an individual's walking or running activities.

Note that all of these general techniques may be used in one mHealth application. It is possible to have personalization, macro-, meso- and micro-tailoring techniques. To score these general techniques the user or participant must be prompted to select an answer or provide input and make decisions in relation to the techniques.

Specific Techniques By Determinant

(Note: The examples given are specific to Cancer Survivorship, but can be adapted for other mHealth behavior techniques or topics.)

Scoring is accomplished by marking the technique with a 1 or 0 in each element or section of the sheet. A "1" indicates that the technique is present in the app, a "0" indicates that it is not present. Personalization and tailoring scoring are provided as additional elements for each major determinant.

Knowledge/Awareness

1. **Provide information about health behavior linkages**.
Basic information about cancer and cancer survivorship, diagnosis, treatment, and/or availability of resources for clinical or non-clinical purposes. If ***Personalized***, the user is prompted to select or provide personal answers about type of cancer or stage of survivorship for example. If ***Tailored,*** the user is required to select actions or elements specific to the intervention and the way information or activities are delivered to them as a result of these choices.

2. **Provide information on action/behavior and consequences.**
Information is provided about the cost/risks/benefits of action or inaction with respect to certain cancer survivorship behaviors. This scoring would also consider risk-communication strategies such as persuasive

communications for example post treatment health screening, smoking cessation or adherence to flu-shot recommendations.

Intention

3. Prompt Intention Formation. The mHealth application includes suggestions for general behavior setting or formulating desired outcomes of a behavior for healthy survivorship, e.g., maintain a healthy weight, exercise regularly, eat 5 fruits and or vegetables daily. It may be sometimes difficult to distinguish this from knowledge or awareness, but coders should look for language that indicates a specific action or activity. Also, note that this technique is different from the actual setting of a goal or behavioral objective to facilitate change or adherence.

Facilitation

4.Provide Instruction. This technique involves telling or showing the user or participant ways to facilitate behavior change. For example explaining "SMART" goal setting, or how to use an app's function to record questions on a mobile phone to ask a provider during an appointment. The function of the instruction must be directly related to the improvement or behavior change, not for general use of the phone or the app. The facilitation may be in the form of written instructions, videos on YouTube that link from the app or images or cartoons that show a step-by-step instruction.

5. Provide materials for education/information. The app provides the cancer survivor with specific materials and information that are suggestions for behavior change. These differ from 1. Knowledge/Awareness in that the education is specific to a behavior change or an action. For example, information on late effects of cancer treatment with prompts of when to contact a healthcare professional. Another example would be educational information about health screenings specific to cancer survivors, for example breast mammograms for female survivors treated with whole body or mantle radiation starting at an early age.

Intention

6. Prompts for Specific Goal Setting. This involves planning and setting a specific goal for what a person would do within a specific time and includes the specific context within which a behavior will be performed. This would include selecting or writing down (micro-tailoring) of a specific goal for example setting a personal goal to "engage in physical activity for 150 minutes each week." Goal setting would include information on when, where, how to act in a specific behavior.

7. Review of general or specific goals. This would involve using the mHealth app in reconsideration of previously set goals or intentions and would require an indication of behavioral performance resulting from self-monitoring or tracking. An example would be review of tracking a goal setting for intake of a specific amount of calories per day or number of minutes of physical activity for a week. Another example might be noting a set of questions to be asked at a healthcare provider appointment regarding levels of pain or emotional distress during a past week or month.

Self-Efficacy

8. Prompt self-monitoring of behavioral goals. The mHealth app suggests that the person record brief notes or keep a journal or diary to record behaviors and actions related to health behaviors. Examples might be a journal of physical activity or pain or distress monitoring and actions taken to alleviate such as meditation or self-talk.

9. Persuasion (verbal or written) The mHealth app delivers messages (may be personalized or tailored) designed to strengthen efficacy/control beliefs related to the execution of target or suggested behaviors. Examples might be often-used successful strategies (e.g., "park at the far end of the parking lot", or "use the stairs instead of the elevator to increase physical activity") or general tips. New beliefs may be induced and/or new information provided to the participant or user to create new control or behavior beliefs.

Social Influence

10. Provide information about peer behavior (Peer passive). The mHealth app provides information about what other cancer survivors do and think in relation to targeted behavior change. This can be provided in the form of written anecdotes YouTube videos and may be presented as interviews or case studies.

11. Provide opportunities for social comparison (Peer active) The mHealth app offers participation in Facebook, Twitter or other social media and networking in which discussion and social comparison may occur. The focus is on providing social reference for the behavior change or activity. Only score this technique when examples of group or peer discussion including personal stories of behavior are shared. For example, a participant cancer survivor shares that “setting my own goals for physical activity and sharing those with my Facebook friends really helped me make my goals.” Or ”writing down my concerns about pain helped me communicate more effectively with my doctor.”

12. Mobilize Social Norms (Important Others) The mHealth app provides exposure to the social norms of important others in relation to a healthy survivorship activity or health behavior change. Important others may be valued and trusted experts such as a recognized healthcare professional, a celebrity cancer survivor or a recognized cancer survivorship researcher or advocate (e.g. Nancy Brinker of Susan G. Komen for the Cure or Cathy Giusti, founder of the Multiple Myeloma Research Foundation.
